# Collaborative Development of an Electronic Portfolio to Support the Assessment and Development of Medical Undergraduates

**DOI:** 10.2196/56568

**Published:** 2024-04-04

**Authors:** Luiz Ricardo Albano dos Santos, Alan Maicon de Oliveira, Luana Michelly Aparecida Costa dos Santos, Guilherme José Aguilar, Wilbert Dener Lemos Costa, Dantony de Castro Barros Donato, Valdes Roberto Bollela

**Affiliations:** 1 Ribeirão Preto Medical School, University of São Paulo, Ribeirão Preto, Brazil; 2 School of Pharmaceutical Sciences of Ribeirão Preto, University of São Paulo, Ribeirão Preto, Brazil; 3 Faculty of Philosophy, Sciences and Letters at Ribeirão Preto, University of São Paulo, Ribeirão Preto, Brazil; 4 Clinical Hospital of the Ribeirão Preto Medical School, University of São Paulo, Ribeirão Preto, Brazil

**Keywords:** e-portfolio, education, health education, learning, medical students, medical school curriculum, medical education, student support, software

## Abstract

This study outlines the development of an electronic portfolio (e-portfolio) designed to capture and record the overall academic performance of medical undergraduate students throughout their educational journey. Additionally, it facilitates the capture of narratives on lived experiences and sharing of reflections, fostering collaboration between students and their mentors.

## Introduction

The Brazilian curriculum guidelines for medical schools incorporate competencies in information technology, emphasizing students’ co-responsibility in acquiring soft skills such as leadership, teamwork, and continuous professional development [1]. The curriculum experience must foster critical and reflective skills [2].

Ribeirão Preto Medical School at University of São Paulo, Brazil (FMRP-USP), is a 72-year-old traditional institution that initiated a curriculum change in January 2023. In this new proposal, we introduced a longitudinal axis and curricular unit called personal and professional development (PPD). The primary objective of PPD is to foster self-reflection on lived experiences, regular self-assessment, and monitoring of the students’ progress in curricular and extracurricular activities, with a mentor’s support.

To support the implementation of the PPD curricular unit, we collaboratively developed a software to serve as the electronic portfolio (e-portfolio) and record the overall academic performance of undergraduate medical students throughout their educational journey. An additional expectation is to encourage and guide teachers to provide and register formative assessments in their disciplines and rotations, and to document their experiences and reflections.

## Methods

The collaborative development of the system involved developers, health educators, and students, which was crucial to ensure that the e-portfolio meets the needs and expectations of all stakeholders. Developers contributed technical expertise for functionality and accessibility, while educators shaped content based on educational principles. Students, as primary users, provided valuable feedback.

The main challenge in developing the e-portfolio was to create an initial set of requirements. With various participants bringing different ideas, there was a multitude of perspectives in the initial phase, which brought fundamental enrichment during development but also increased the difficulty of integrating all perspectives.

These challenges were overcome with Scrum [3] integrated with socio-technical research methodology to facilitate the collaborative environment. We implement Scrum practices, such as daily 5-minute meetings and biweekly 30-minute sprint reviews, ensuring incremental and continuous deliveries and communication between the development team and stakeholders, mainly regarding system development. Additionally, we integrated the socio-technical research methodology [4] into SCRUM, aiming to understand the software requirements as well as the various social and technological factors involved.

Regarding software development technologies, we used HTML, CSS, PHP, and the MySQL database management system.

## Ethics Approval

The study received approval from the research ethics committee of the Clinical Hospital of FMRP-USP (CAAE: 67577523.1.0000.5440).

## Results

**Figure 1.**
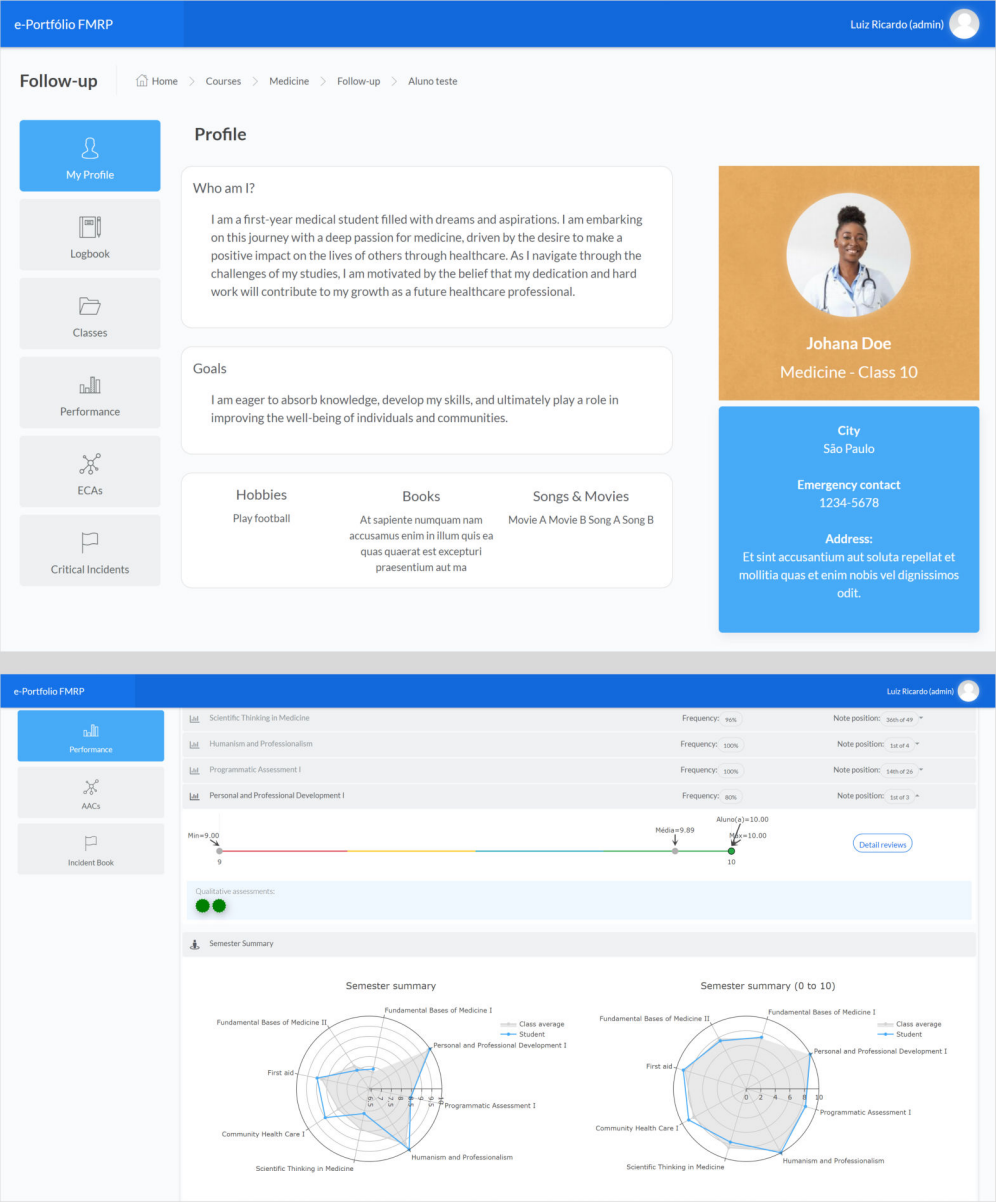
Profile and performance report of the medical student in the electronic portfolio (e-portfolio). (A) Profile created by the student in the e-portfolio. (B) Student’s performance in various subjects is presented in relation to the radar chart: the blue line represents a comparison with the cohort mean (depicted by the gray area).

The e-portfolio utilizes a web application architecture (Figure 1). Initially, we developed a structure to manage the registration of all the programs within the medical school, different curricular units, and offerings. We created a registration module for students and faculty members, allowing those to act as mentors, teachers, and discipline coordinators. Additionally, e-portfolio enables the recording of direct observed assessments in clinical settings, using preregistered forms based on methods such as mini-clinical evaluation exercise (Mini-CEx) [5], 360-degree assessment [6], One-Minute Preceptor, direct observation of procedural skills (DOPS), and case-based discussion/chart-stimulated recall (CBD/CSR) [7].

**Figure 2.**
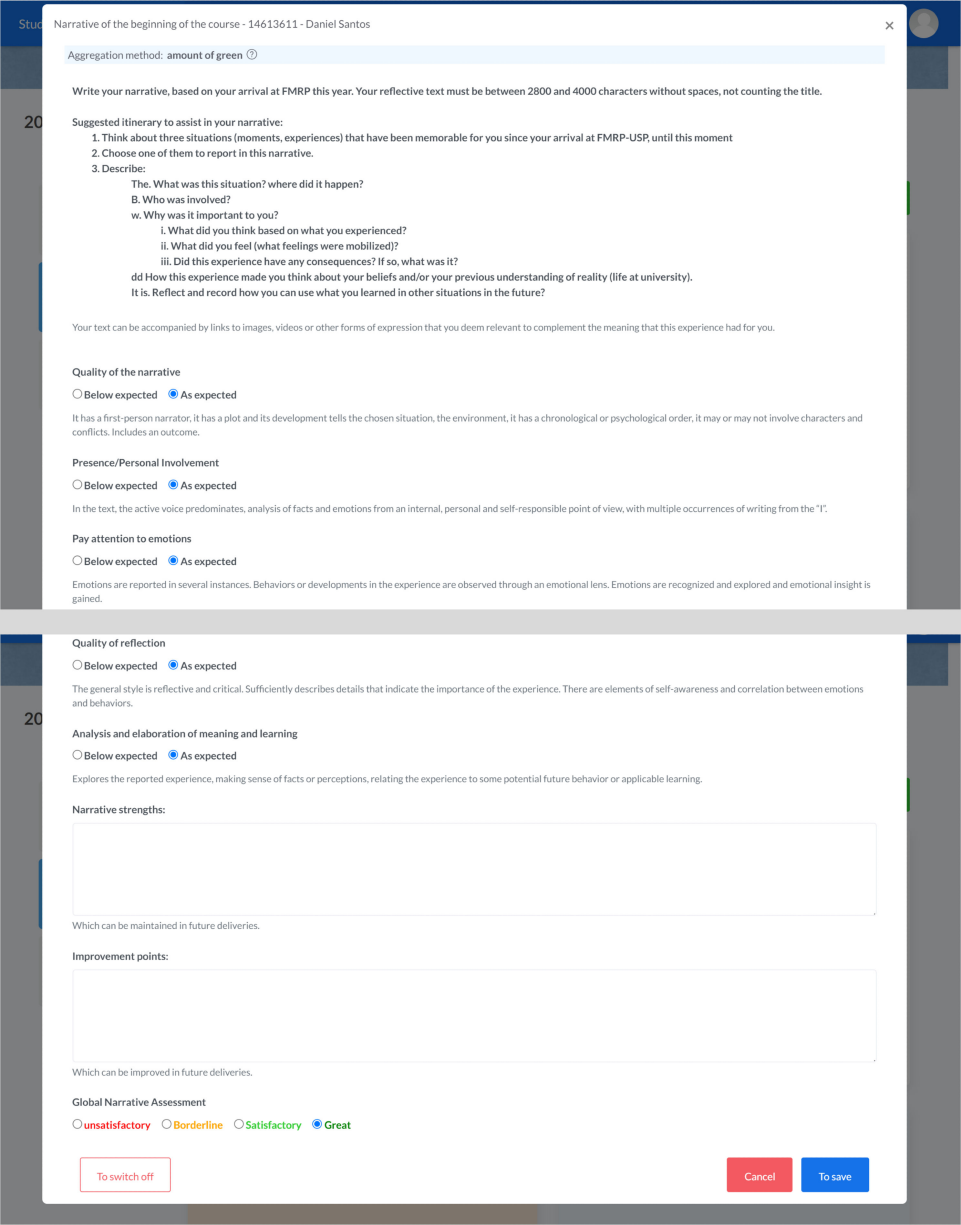
Evaluation form in the electronic portfolio (e-portfolio) with narratives and the adapted REFLECT rubric to guide the medical student and the mentor.

For narratives in medicine [8], there is a specific form to guide students on how to report a lived experience followed by a meaningful reflection, based on the REFLECT rubric for assessing reflective writing [9] (Figure 2).

Students are allowed to fill in data in their private profile (Figure 1), access their disciplines and received assessments, respond to formative assessments, record significant events for their education, check and compare their performance with their cohort, register extracurricular activities, and consult critical incidents recorded.

e-Portfolio enables students, discipline coordinators, and members of the student assessment committee to track assessments and feedback received, providing a longitudinal and progressive view of the student’s cognitive, psychomotor (skills), and attitudinal development (Figures1  2).

## Discussion

This work presents the successful development of an e-portfolio at FMRP-USP. The e-portfolio is continuously enhanced and updated, and it is currently in a state suitable for use in a pilot study. The use of similar tools has been recognized for stimulating personal reflection, fostering collaboration, and strengthening digital literacy among students, encouraging active participation in the learning process [10].

The application of Scrum offered an adaptable framework, promoting efficient collaboration among stakeholders. Additionally, socio-technical research methods, such as qualitative interviews involving in-depth conversations with individuals or groups to explore their experiences related to technology, provided valuable insights into the needs and dynamics of end users in the educational context. The use of Scrum with socio-technical research methods enables a more integrated, collaborative, and reflective approach during development.

### Future Steps

We intend to evaluate e-portfolio usability, effectiveness, acceptance, and satisfaction in practical contexts with the objective of consistently enhancing the system and its outcomes.

## Abbreviations

CAPES: Coordenação de Aperfeiçoamento de Pessoal de Nível Superior (Coordination for the Improvement of Higher Education Personnel)
CBD/CSR: case-based discussion/chart-stimulated recall
CNPq: Conselho Nacional de Desenvolvimento Científico e Tecnológico (National Council for Scientific and Technological Development)
DOPS: direct observation of procedural skills
e-portfolio: electronic portfolio
FMRP-USP: Ribeirão Preto Medical School, University of São Paulo
Mini-CEx: mini-clinical evaluation exercise
PPD: personal and professional development
